# Supplementary material for: Educational technologies for teaching hand hygiene: Systematic review
Source: PLoS One. 2024 Jan 16;19(1):e0294725. doi: 10.1371/journal.pone.0294725 (PMC10790983; doi:10.1371/journal.pone.0294725)
Supplement: Attachment — Submitted filename: Response to reviewers .pdf [file pone.0294725.s002.pdf]

UNIVERSITY OF SÃO PAULO
AT RIBEIRÃO PRETO COLLEGE OF NURSING
Avenida Bandeirantes, 3900 - Ribeirão Preto - São Paulo - Brasil - CEP: 14040-902
Phones: 55 16 3315.3393 - e-mail: eerp@usp.br - www.eerp.usp.br

Dear Maria José Nogueira, Ph.D.
Academic Editor
PLOS ONE

On behalf of all authors, I inform you that we proceeded with a thorough review of the manuscript based on the reviewers' observations and suggestions. We emphasize that all manuscript changes are marked with yellow highlights. In addition to the reviewers' requests, we also revised Table 6 to present the risk of bias assessment as recommended by JBI. The responses to the comments are described below:

Reviewer #1:
Congratulations on the choice of the topic for the systematic review.
Abstract
Describe the main objetive of the study; Explain how the review was done; Summarize the most important results and their significance; Not exceed 300 words. At this point, it would be importante to clarify which target population was selected for the systematic review, as well as in which care contexts.
Response: We appreciate and accept the reviewer's suggestion. The main objective of the study was described in the Abstract: "To gather available scientific evidence on technologies used to teach hand hygiene to professional populations and lays involved in health care in the hospital setting", clarifying the target population and the care context of the systematic review, which was professional populations and lays involved in health care in the hospital setting. We also better explained how the review was done.

Introduction
At this point, it would be importante to demonstrate the worldwide of cross infections caused by improper hand hygiene or incorrect hand hygiene, whith a focus on the risks of mortality and morbidity.
Response: We appreciate and accept the reviewer's suggestion. To accomplish this request, we added the second and third paragraphs of the Introduction section: "The prevention of HAIs should

be a priority action for healthcare organizations. According to the WHO, out of 100 patients, seven will acquire at least one healthcare-associated infection during their hospital stay, the risk doubles and may even increase 20 times in low- and middle-income countries. The sicker and more fragile patients become, the greater becomes the risk of HAI and their deathly consequences [3]. Infection prevention and control interventions can reduce infections in healthcare by 70%, specially HH programs can reduce the risk by more than half of dying as a result of infections, and also reduce associated long-term complications and healthcare costs [4]."

Materials and Methods

1. Clarify what type of descriptors were used for the DeCS/MeSH search in the research.

Response: We appreciate and accept the reviewer's suggestion. The DeCS/MeSH descriptors used for the search of this systematic review was described in page 4, and was: "technology", "health technology" and "hand hygiene".

2. Clarify inclusion and exclusion criteria regarding the population (children, adults, elderly?) and the contexts. If all were included, state this explicitly.

Response: We appreciate and accept the reviewer's suggestion. Inclusion and exclusion criteria were rewritten on page 5 as: "This review included studies reporting primary, quantitative research findings identifying the use of educational technologies about HH among populations of health professionals, patients, family members and visitors involved in health care in the hospital setting, of any age group, published in English, Portuguese or Spanish, no data limit. Excluded from this review were case reports, case series, secondary studies (other reviews), editorials, letters to the editor, books, book chapters, guidelines, expert opinion articles, experience reports, conference proceedings and abstracts, dissertations and theses, in addition to studies outside the scope of this review", clarifying that the population was health professionals, patients, family members and visitors, of any age group, in the context of health care in the hospital setting.

3. Clarify the ethical issues inherent in a systematic review.

Response: We appreciate and accept the reviewer's suggestion. Since the review did not involve human participants, it did not require ethical committee approval. However, the authors conducted

this review with a strong emphasis on transparency and reproducibility, avoiding bias, and ensuring a comprehensive and fair evaluation. This was clarified on page 6.

Conclusion

Given the richness of the topic, it could be improved by highlighting the conclusions obtained in research of this nature. It would be important to demonstrate what limitations/difficulties were encountered in this work and what suggestions the authors have for future research.

Response: We appreciate and accept the reviewer's suggestion. We highlighted the conclusions obtained in this systematic review adding the paragraph: “The presentation of educational content on HH through technological resources such as mobile devices, virtual reality, and audio-visual reminders, among others, facilitates HH teaching in sharing and accessing information, and helping patients and family members learn the hand washing procedure. Furthermore, they are efficient tools for improving HHC rates among health professionals and open up opportunities for repetitive, efficient, and evidence-based learning”.

We demonstrated the limitations/difficulties encountered in this work with the paragraph: “As a limitation, the small number of studies included in this review had different populations, interventions, and outcomes, due to these it was not possible to perform a metanalysis. Moreover, they have a vulnerable methodological quality, which can compromise generalization of the results to other contexts. However, the results presented in this review may support the development of new studies with other designs on the theme and its practical relevance.”

We added the suggestions for future research with the paragraph: “The scientific literature gathered in this review also highlights the importance of encouraging the development of studies aiming to develop technologies for teaching such practice to the entire population. New studies with robust methodological approaches are needed to establish which technological tool for teaching HH is the most effective to be implemented”.

Reviewer #2:

1) A Systematic Review (SR) of prevalence must include cross-sectional and longitudinal studies. I missed the inclusion of cohort studies to establish a causal relationship between the outcome and the exposure factor (non-hand hygiene). Therefore, for this relationship to be observed, data must be collected at different moments over time.

Response: Thank you for your comment. We did not plan to conduct a prevalence review, as this review did not assess how many people did HH (yes- hand hygiene) and how many people did not (non-hand hygiene). Therefore, quasi-experimental studies and randomized clinical trials, were included. The studies addressed the use of technologies to teach professionals or lay populations (such as family members, visitors, relatives, and patients) about hand hygiene and to support and encourage their development for this purpose, as proposed in the objective of our review. Finally, given the heterogeneity of the included studies, we did not perform a meta-analysis of the obtained data.

2) Prevalence SRs, when well designed and rigorously conducted, produce potential guiding and recommendation results for managers and health professionals in the development of specific policies for conditions that most affect and impact a given population. It is necessary for RS authors to be able to identify the different types of prevalence that are being presented in the included studies.

Response: Thank you for your comment. In this review, we did not intend to conduct a prevalence review with meta-analysis. While the topic of hand hygiene is broad and comprehensive, the use of technologies for teaching hand hygiene is still in its early stages, which would preclude analyses like prevalence meta-analyses. For this reason, we adhered to the objective of our review and included studies that addressed educational strategies for teaching hand hygiene, presenting the results in a descriptive manner.

3) Describe in a clear and detailed way the items of the JBI tool that were most important for the analysis of methodological quality. Which questions had the greatest weight when considering the study with greater or lesser methodological quality?

Response: We appreciate and accept the reviewer's suggestion. To accomplish this request, we added three paragraphs below the subitem “Critical appraisal of the methodological quality” on page 10.

4) The discussion was approached in a very general way. Relate the findings of the studies included in the SR with the evidence available in the literature.

Response: We appreciate and accept the reviewer's suggestion. To accomplish this request, we added and rewrite some paragraphs of the Discussion section, corroborating our findings with data from the literature.

Renata Cristina de Campos Pereira Silveira

RN PhD Associate Professor

University of São Paulo

Ribeirão Preto College of Nursing
